# The blood of healthy individuals exhibits CD8 T cells with a highly altered TCR Vß repertoire but with an unmodified phenotype

**DOI:** 10.1186/1479-5876-9-S2-P4

**Published:** 2011-11-23

**Authors:** Nicolas Degauque, Françoise Boeffard, Yohann Foucher, Caroline Ballet, Jean-Paul Soulillou, Sophie Brouard

**Affiliations:** 1 INSERM, UMR643, Nantes, France; 2 Université de Nantes, Faculté de Médecine, Nantes, France; 3 INSERM, EA 4275, Nantes, France

## 

CD8 T cell clonal expansions (TCE) have been observed in elderly, healthy individuals as well in old mice, and have been associated with the ageing process. Both chronic latent and non-persistent viral infections have been proposed to drive the development of distinct non-functional and functional TCE respectively. Biases in TCR Vβ repertoire diversity are also recurrently observed in patients that have undergone strong immune challenge, and are preferentially observed in the CD8 compartment. Healthy adults can also exhibit CD8 T cells with strong alterations of their CDR3 length distribution. Surprisingly, no specific investigations have been conducted to analyze the CD8 T cell repertoire in normal adults, to determine if such alterations in TCR Vβ repertoire share the features of TCE. In this study, we characterized the phenotype and function of the CD8 population in healthy individuals of 25-52 years of age. All but one of the EBV-positive HLA-B8 healthy volunteers that were studied were CMV-negative. Using a specific unsupervised statistical method, we identified Vβ families with altered CDR3 length distribution and increased TCR Vβ/HPRT transcript ratios in all individuals tested. The increase in TCR Vβ/HPRT transcript ratio was more frequently associated with an increase in the percentage of the corresponding Vβ^+^ T cells than with an absence of modification of their percentage. However, in contrast with the previously described TCE, these CD8^+^ T cells were not preferentially found in the memory CD8 subset, they exhibited normal effector functions (cytokine secretion and cytotoxic molecule expression) and they were not reactive to a pool of EBV/CMV/Flu virus peptides. Taken together, the combined analysis of transcripts and proteins of the TCR Vβ repertoire led to the identification of different types of CD8^+^ T cell clone expansion or contraction in healthy individuals, a situation that appears more complex than previously described in aged individuals.

